# Characterization of long non-coding RNAs during compatible and incompatible pollination in *Arabidopsis thaliana*

**DOI:** 10.3389/fpls.2026.1868651

**Published:** 2026-07-29

**Authors:** Nischay Patel, Nilesh D. Gawande, Subramanian Sankaranarayanan

**Affiliations:** 1 Department of Biological Sciences and Bioengineering, Indian Institute of Technology Kanpur, Kanpur, Uttar Pradesh, India; 2 Department of Biotechnology, School of Sciences, Woxsen University, Hyderabad, Hyderabad, Telangana, India; 3 Department of Biological Sciences and Engineering, Indian Institute of Technology Gandhinagar, Palaj, Gujarat, India

**Keywords:** *Arabidopsis thaliana*, compatible/incompatible pollination, long non-coding RNAs (lncRNAs), post-pollination transcriptomics, regulatory networks

## Abstract

Long non-coding RNAs (lncRNAs) have emerged as critical players in plant development and stress responses, yet their involvement in pollination responses is largely unknown. To address this gap, we identified and characterized lncRNAs and their predicted cis-acting, trans-acting, and miRNA-mediated possible regulatory interactions during both compatible and incompatible pollination in *Arabidopsis thaliana*. Leveraging publicly available datasets, we analyzed expression profiles at 10 and 60 minutes post-pollination. We identified 1,073 novel and 3,422 annotated lncRNA loci, with 1,002 novel and 985 annotated, respectively, showing detectable expression after filtering. Differential expression analysis identified 12 potential lncRNA loci at 10 min and 32 lncRNA loci at 60 min post-pollination. Further investigation predicted 9 potential cis-targets, 103 trans-targets, and 144 miRNA-mediated targets, many of which were enriched in pathways related to stress, defense, and self-incompatibility in Gene Ontology analysis. Notably, the predicted regulatory landscape is more active at 60 minutes than at 10 minutes post-pollination. These findings provide a robust framework and resource to facilitate future functional studies of these potential lncRNAs during pollination.

## 1 Introduction

Long non-coding RNAs (lncRNAs) are characterized by transcripts longer than 200 base pairs that do not encode proteins. Plant genomes contain tens of thousands of lncRNAs that originate from coding regions, introns, or intergenic regions, and are usually transcribed by RNA polymerase II from both the sense and antisense strands. However, other polymerases, such as RNA polymerase IV and V, also produce lncRNAs in plants (Wierzbicki et al., 2021). LncRNAs play a key role in the regulation of molecular and biological processes, as well as cellular and developmental functions, through RNA-dependent mechanisms.

LncRNAs exhibit diverse regulatory functions, determined by their sequence composition, structure, and interaction partners (Wang and Chang, 2011; Wierzbicki et al., 2021; Herman et al., 2022). In plants, lncRNAs are involved in various cellular and molecular mechanisms, including chromatin modification, transcriptional regulation, RNA processing, mRNA stability, and translational control (Ariel et al., 2015; Quinn and Chang, 2016; Zhao et al., 2024; Shen et al., 2026). LncRNAs exhibit distinct characteristics, including generally lower sequence conservation, lower expression levels, and pronounced treatment- and tissue-specific expression patterns compared to protein-coding genes. Beyond these features, lncRNAs often play important regulatory roles (Ulitsky, 2016; Golicz et al., 2018).

In plants, lncRNAs are categorized based on their relative genomic location to protein-coding genes. Sense lncRNA overlaps with the exonic regions of protein-coding genes on the same strand, while antisense lncRNAs are transcribed from the opposite strand of protein-coding genes. Intronic lncRNAs are transcribed from intronic regions of protein-coding genes, while intergenic lncRNAs are produced from regions between the annotated genes. LncRNAs can function either by acting near the genomic locus from which they are transcribed (cis) or by regulating distant targets (trans), including through miRNA-mediated regulatory interactions such as target mimicry, where lncRNAs bind miRNA and block their interaction with target mRNA (Franco-Zorrilla et al., 2007; Gil and Ulitsky, 2020).

In flowering plants, successful fertilization determines the future of seed production. Various signaling events happening during communication between compatible male reproductive tissue (pollen) and the female tissue (the pistil) have a critical role in reproduction. During pollination, a desiccated pollen grain lands on the stigma papillae cells of the pistil and shuts down the Reactive Oxygen species (ROS) signaling pathway at the stigmatic surface, resulting in further events such pollen hydration and germination, which further leads to the formation and elongation of the pollen tube through the stigmatic cell wall, enabling it to reach the ovary and release the male gametes to the ovule for fertilization (Jamshed et al., 2023). Plants distinguish between compatible and incompatible pollen, which helps them achieve reproductive success and maintain genetic diversity (Muñoz-Sanz et al., 2020).

In Brassicaceae species such as Arabidopsis and Brassica, compatible pollination involves several steps, including pollen hydration, germination, and pollen tube growth. In contrast, incompatible pollination leads to rapid pollen rejection at the stigma surface itself (Hiscock, 2002; Chae and Lord, 2011). Self-incompatibility responses are highly specific and are mediated by interaction between the stigma-expressed S-locus receptor kinase (SRK) and the pollen coat-derived S-locus cysteine-rich protein (SCR/SP11) in Brassicaceae, resulting in activation of signaling cascades that lead to pollen germination inhibition (Takayama et al., 2000; Yamamoto and Nishio, 2014; Bhalla et al., 2025a). The interaction of either a self or non-self pollen with stigmatic papillae triggers a distinct transcriptional response within minutes of pollination, ultimately determining whether the pollen is accepted or rejected (Sankaranarayanan et al., 2013; Zhang et al., 2017; Kodera et al., 2021). This selective process is biologically significant as it is a key determinant of fertilization efficiency and seed set (Ferrer et al., 2009). Therefore, the transcriptional reprogramming in pollen and stigma during compatible and incompatible pollination includes various cellular responses, signaling events, vesicle trafficking, cytoskeletal remodelling, and metabolic adjustments that determine pollen acceptance or rejection (Iwano et al., 2007; Kodera et al., 2021).

Since *Arabidopsis thaliana* is a self-compatible species, Kodera et al. (2021) achieved an experimental self-incompatibility by introducing the *AlSRK14* construct into the Col-0 accession (Col-0/*SRK14*) and the *AlSCR14* construct into the C24 accession (C24/*SCR14*). Further, pollination of Col-0/*SRK14* stigmas with C24 pollen resulted in a compatible interaction, whereas pollination with C24/*SCR*14 pollen resulted in an incompatible interaction (Kodera et al., 2021). At the molecular level, transcriptome analysis in *Arabidopsis* and *Brassica napus* demonstrate that pollination responses are highly dynamic and time-dependent. Early transcriptional changes are detectable as soon as 10 minutes after pollination and diverge into distinct expression profiles by 60 minutes, distinguishing compatible from incompatible interactions (Sankaranarayanan et al., 2013; Zhang et al., 2017; Kodera et al., 2021). In the stigmas, during the compatible pollination, genes involved in MAPK signaling and plant-pathogen interaction pathways such as *Mitogen-Activated Protein Kinase 3* and *6* (*MKK3/6*) and *WRKY Transcription Factor 22* and *33* (*WRKY22/33*), are rapidly induced within 10 min, with particularly strong upregulation of *MPK3* and *WRKY33*. In contrast, incompatible pollination showed minimal expression of 4 stigma-specific genes at 10 minutes; however, by 60 minutes, 104 genes were upregulated, indicating a delayed yet extensive transcriptional response characteristic of incompatibility (Kodera et al., 2021). The incompatibility-related genes upregulated at 60 min include receptor-like kinases *CRK41* and *CRK31*, and factors associated with endocytosis, secretion, and defense-related processes, such as *FLOT1*, *ROH1*, and *MLO12*. In pollen, compatible interactions are characterized by the upregulation of pollen-expressed and pollen tube associated genes, including *CHX21*, implicated in pollen tube guidance, as well as a receptor-like cytoplasmic kinase *(RLCK)* family gene at 60 min, both showing higher fold changes compared to incompatible interactions at the same time point (Kodera et al., 2021). These varied transcriptional changes across time points highlight the significance of time-dependent sample collection in capturing early versus late regulatory molecular signatures (Sankaranarayanan et al., 2013; Zhang et al., 2017).

In plant growth regulation, long non-coding RNAs (lncRNAs) play diverse functional roles, including antisense and splicing-associated regulation. Notable examples such as COOLAIR and ASCO contribute to the maintenance of shoot apical meristem integrity by modulating transcriptional programs, alternative splicing, and auxin-responsive gene expression (Marquardt et al., 2014; Rosa et al., 2016; Rigo et al., 2020). Another auxin-responsive lncRNA, APOLO, functions in roots to regulate lateral root and root hair development by translating auxin signals into chromatin-level modifications, thereby reprogramming transcriptional networks that control auxin distribution and root patterning (Ariel et al., 2014, 2020). Additionally, the antisense lncRNA associated with DOG1 regulates seed dormancy through chromatin-mediated transcriptional control (Fedak et al., 2016). Beyond their established roles in plant development and hormone signaling, these findings suggest that lncRNAs may also contribute to reproductive processes, particularly through transcriptional reprogramming during pollen-pistil interactions.

In plant reproduction, functional evidence supporting the role of lncRNAs in pollen fertility has been demonstrated in rice, where disruption of the lncRNA LDMAR leads to photoperiod-sensitive male sterility, directly linking lncRNA regulation to reproductive success (Ding et al., 2012). High-throughput sequencing analysis of anthers across various developmental stages in *A. thaliana* identified 1,283 lncRNAs, with predicted functions including roles as miRNA precursors, endogenous target mimics, or natural antisense transcripts of primary miRNA (Zhou et al., 2025). Similarly, in *Brassica rapa*, 12,051 lncRNAs were profiled across five pollen stages, including pollen mother cell, tetrad, uninucleate pollen, binucleate pollen, and mature pollen (Huang et al., 2018). Environmental cues further influence lncRNA activity during reproductive development; for example, heat stress during pollen development in wheat induces 5,482 lncRNAs associated with predicted cis- and trans-target genes involved in heat response, protein folding, abiotic stress signaling, and jasmonic acid biosynthesis pathways (Babaei et al., 2024).

Despite the growing body of transcriptomic data, most studies have focused primarily on protein-coding genes, leaving the roles of non-coding regulators such as lncRNAs during pollination relatively underexplored. Although lncRNAs are increasingly recognized as key regulators of gene expression, their specific contributions to pollination and reproductive responses remain poorly understood.

In this study, we identified and characterized lncRNAs by reanalysis of the transcriptomic datasets generated from pollinated Arabidopsis stigmas under compatible and incompatible pollination conditions at 10 and 60 min, respectively (Kodera et al., 2021). The identified lncRNAs were classified based on their genomic context, specific expression patterns, and differential expression at 10 min and 60 min after pollination. Furthermore, we predicted cis- and trans-regulatory interactions between lncRNAs and protein-coding genes, constructed lncRNA-miRNA-mRNA regulatory networks, and performed functional enrichment analysis to infer the potential biological roles. Together, these findings provide new insights into the spatial and temporal dynamics of lncRNA expression in stigma and pollen tissues during compatible and incompatible pollination. This study also highlights the possible interactions between lncRNAs and other regulatory miRNAs, elucidating the complex regulatory networks that underpin pollination-associated transcriptional reprogramming.

## 2 Materials and methods

### 2.1 Data retrieval and quality control

The raw RNA-seq data for compatible and incompatible pollination at two time points (10 min and 60 min post-pollination were retrieved from a publicly available dataset (Accession no: SRP154565, Kodera et al., 2021) in the Sequence Read Archive (SRA) at the National Center for Biotechnology Information (NCBI) database. The dataset included the transcriptomes of pollinated stigmatic tissues from compatible pollination (Col-0/*SRK14* x C24) and incompatible pollination (Col-0/SRK14 x C24/SCR14) (Kodera et al., 2021) at 10 min (t = 10) and 60 min (t = 60) post-pollination (**Supplementary Table S1**). The reference genome assembly (TAIR10, GCA_000001735.1) and gene annotation files (Araport11) were obtained from the Ensembl Plants Release 62. We used the same reference files throughout the transcript assembly, lncRNA identification and all downstream analysis. The details for the samples and reference annotation file sources are provided in **Supplementary Tables S1**, **S2**.

RNA-seq data quality and replicate consistency were assessed using FastQC (Andrews, 2010) and principal component analysis (PCA) of variance-stabilized expression values. This analysis was further complemented by a Euclidean distance-based metric calculated on VST-transformed data to assess the relatedness among biological replicates (**Supplementary Table S3**; **Supplementary Figure S1**). In summary, pairwise euclidean distances were assessed across all samples. For each replicate, the mean distance was calculated with respect to other replicates belonging to the same pollination condition. To identify divergent replicates, mean distance of each replicate was normalised by the median distance, generating a ratio of mean to median. After evaluating both PCA plots and replicate distances, one divergent replicate in each condition at each time-point (10_compatible3, 10_incompatible4, 60_compatible4, and 60_incompatible2) was removed. The final dataset included three independent biological replicates for pollinated stigmas under compatible and incompatible pollination, collected at 10 and 60 min post-pollination.

### 2.2 Transcriptome reconstruction and lncRNA identification pipeline

The adapter sequences and low-quality bases in the raw reads were trimmed using Trimmomatic (Bolger et al., 2014). The library strandedness for the RNA-seq samples was determined using an online tool (https://github.com/signalbash/how_are_we_stranded_here) (Signal and Kahlke, 2022). All samples showed a consistent Reverse-stranded (RF) (fr-firststrand) library orientation, with 99.5-99.6% of explainable reads supporting this configuration (**Supplementary Table S4**). Accordingly, reverse-stranded settings were used for downstream transcript assembly and expression analysis. High-quality reads were aligned to the *A. thaliana* reference from TAIR10 genome assembly (Araport11 gene annotation) using HISAT2 (Kim et al., 2015). For each sample, per-sample transcript assemblies were generated using StringTie (Pertea et al., 2015; Kovaka et al., 2019) in reference guided mode. Six independent assemblies per time point were merged into a unified transcriptome annotation with StringTie-merge, and the merged transcriptome was compared to the Araport11 reference annotation using GffCompare (Pertea and Pertea, 2020) to classify transcript structures. Transcript and gene identifiers were reconciled by integrating gffcompare annotation and tracking files. Reference-matching transcripts (class_code “=“ or “c”) were linked to original Araport11 annotated identifiers, while all other transcripts were assigned standardized TCONS (transcript-level) and XLOC (locus-level) identifiers, which were incorporated into the final GTF annotation file. Further, the merged annotation in GTF format was converted to BED12 format, and transcript sequences were extracted from the TAIR10 genome assembly (Araport11 gene annotation) using the BEDTools getfasta function (Quinlan and Hall, 2010). Transcripts shorter than 200 nucleotides were excluded from further analysis. The protein-coding genes, small non-coding RNAs, and non-translating CDS were excluded from further analysis. Transcripts annotated as lncRNA in the Araport11 annotation were considered as annotated lncRNAs. Other transcripts, including those annotated as different RNA biotypes in Araport11 or absent from the Araport11 annotation, were treated as putative novel lncRNA candidates and were subjected to a multi-step filtering pipeline.

The resulting novel lncRNA set thus consisted of two types: (i) transcripts originating from annotated genomic loci that were not previously classified as lncRNAs in Araport11 (with AT-locus identifiers), and (ii) newly assembled transcripts that were not represented in Araport11 (with XLOC and TCONS identifiers). Throughout this study, novel lncRNAs with AT-locus identifiers should therefore be interpreted as reclassified Araport11 transcripts that were identified as lncRNAs by our pipeline, while novel lncRNAs with XLOC and TCONS identifiers represent novel transcripts absent from the Araport11 annotation. This approach effectively identified previously unannotated lncRNAs while discarding well-characterized protein-coding genes and small non-coding RNAs.

#### 2.2.1 Coding potential assessment

The coding potential of unannotated transcripts was determined using three independent tools that included CPC2, CPAT, and LncFinder. The cutoff considered for these tools included CPC2 (coding probability<0.5) (https://github.com/gao-lab/CPC2_standalone) (Kang et al., 2017), CPAT (coding probability<0.46) (https://github.com/liguowang/cpat) (Wang et al., 2013), and LncFinder (https://cran.r-project.org/web/packages/LncFinder/index.html) (coding potential<0.5) (Han et al., 2019). Plant-specific pre-trained models and corresponding cutoff values as defined in the Plant-LncPipe framework were used for CPAT and Plant LncFinder (https://github.com/xuechantian/Plant-LncRNA-pipline) (Tian et al., 2024). Transcripts that passed all three tools criteria were retained as novel candidate lncRNAs.

#### 2.2.2 Protein domain filtering

To further refine the selection of potential lncRNAs, novel candidate lncRNAs were screened against the Pfam database (Mistry et al., 2021) using PfamScan (HMMER) https://github.com/aziele/pfam_scan). Transcripts that contained Pfam domain hits with domain E-values ≤ 1e-3 were considered protein-coding and excluded from subsequent analysis.

#### 2.2.3 Construction of the final lncRNA set

The resulting high-confidence novel lncRNAs were integrated with previously annotated lncRNAs to create a final lncRNA set. From transcript-level annotations, lncRNA loci were defined by grouping lncRNA transcripts based on their genomic coordinates; any locus that generated at least one lncRNA transcript was classified as a lncRNA locus.

### 2.3 Classification of lncRNAs based on their genomic positions

The novel lncRNAs were subjected to the FEELnc (Flexible Extraction of Long non-coding RNAs) classifier (https://github.com/tderrien/FEELnc) (Wucher et al., 2017), which assigns lncRNAs to genomic categories based on their positional relationship to annotated protein-coding transcripts. Each lncRNA was first classified as either genic (overlapped with an annotated gene) or intergenic (located between annotated genes). Genic lncRNAs were further subclassified based on their overlap with coding genes (exonic, intronic, overlapping, containing, or nested), whereas intergenic lncRNAs were categorized based on their relative orientation and genomic position relative to neighbouring genes (divergent, convergent, or same-strand, and upstream or downstream).

### 2.4 Transcript expression quantification and classification

Transcript-level expression quantification was performed using Kallisto (Bray et al., 2016) using the merged transcriptome as the reference index. Expression levels for samples were calculated as transcripts per million (TPM). Transcripts and coding genes with low expression were filtered using mean-variance trend analysis. The inspection of the mean-variance relationship using LOWESS smoothing indicated increased variability at TPM ≤ 0.05 (**Supplementary Figure S2**). The RNA-seq dataset provided substantial sequencing depth for transcript detection, with the 12 samples retained for downstream analysis having an average sequencing depth of 46.0 million cleaned paired-end reads per sample with samples ranging from 36.6-50.8 million read pairs and an average of 90.6 million mapped reads per sample following alignment to the TAIR10 genome assembly (Araport11 gene annotation) (**Supplementary Table S5**). Considering the lower expression levels of lncRNAs compared to messenger RNAs (mRNAs) (Grammatikakis and Lal, 2022), a permissive cutoff for expression of lncRNA transcripts was set at TPM > 0.05.

To ensure accurate expression analysis and minimize artifacts from low or inconsistent transcript detection, an expression-based filtering step was applied before differential expression analysis. An lncRNA was considered expressed at time points (t = 10 or t = 60 min) if at least one transcript had TPM > 0.05 in at least two of three biological replicates in either the compatible or incompatible condition. Among the expressed lncRNAs, condition-specific expression was defined using a stricter criterion: lncRNA transcripts were classified as condition-specific if they had TPM > 0.1 in at least two out of three replicates in one condition and no detectable expression (TPM = 0) in all three replicates of the other condition at the same time. The expressed lncRNA genes and their corresponding transcripts were retained for differential expression analysis.

### 2.5 Differential expression analysis of lncRNAs and mRNAs

Gene level counts and transcripts per million (TPM) values were generated from transcript-level estimates using the tximport R package with the lengthScaledTPM method. Differential expression analysis of expressed lncRNA genes and protein-coding mRNAs was conducted using DESeq2 (read count threshold ≥ 10) (Love et al., 2014). Fold change was calculated as, log2(TPM Incompatible Sample)/(TPM Compatible Sample)). Statistical significance was assessed with Wald tests, and p-values were adjusted for multiple testing using the Benjamini-Hochberg false discovery rate (FDR) (Haynes, 2013). Differential expression was considered significant for adjusted p-values < 0.1 and absolute fold change ≥ 1. Subsequent functional analysis and biological interpretation were performed at the gene (locus) level.

### 2.6 *In silico* validation of predicted differentially expressed and novel lncRNAs

To validate the identified DE lncRNAs in this study, we assessed the expression of these lncRNAs in independent pollen and stigma tissue datasets using Arabidopsis RNA-seq Database (AthRDB; https://plantrnadb.com/athrdb/), which indexes more than 20,000 publicly available *A. thaliana* RNA-seq libraries. The details for the pollen and stigma datasets used for the confirmation of DE lncRNAs are provided in **Supplementary Table S6**. Additionally, we further verified the DE and novel lncRNAs against three independent *Arabidopsis thaliana* lncRNA catalogues, including PLncDB v2.0 (https://www.tobaccodb.org/plncdb/, Jin et al., 2021), CANTATAdb 3.0 (http://cantata.amu.edu.pl/, Szcześniak and Wanowska, 2024), and GreeNC v2.0 (http://greenc.sequentiabiotech.com/wiki2/Main_Page, Di Marsico et al., 2022). These databases provide an lncRNA catalogue under plant developmental and stress conditions identified by RNA-seq or qRT-PCR/RT-PC or Microarray. The DE and novel lncRNAs nucleotide sequences were used to search against these databases using BLASTN search. A lncRNA hit was considered as significant when satisfied the criteria of E-value ≤ 1e-10, bit score ≥ 50, query coverage ≥ 55%, and sequence identity ≥ 80% (Lu et al., 2017).

### 2.7 Identification of cis and trans targets of lncRNAs

Cis target genes of lncRNAs were identified based on genomic proximity and expression correlation, focusing only on differentially expressed (DE) lncRNAs. DE protein-coding mRNAs were treated as potential DE lncRNA targets. For each lncRNA, the ten nearest protein-coding genes located within 100 kb upstream and downstream of the lncRNA locus were identified using BEDTools (Quinlan and Hall, 2010). Overlapping protein-coding genes were also included. Genes that did not fall within a 100 kb were considered as potential trans targets. Each timepoint (10 min and 60 min) in this study consisted of n = 6 samples (3 compatible pollination + 3 incompatible pollination replicates). To further consider a lncRNA-mRNA pair for correlation analysis, only pairs in which both lncRNAs and mRNAs exhibited expression values of TPM > 0.05 in at least 4 out of 6 samples were retained. To refine the lncRNA-mRNA associations, Pearson correlation and Spearman correlation coefficients were calculated using log_2_(TPM + 1) transformed values and their p-values were calculated for each candidate lncRNA-mRNA pair using the R stats package (cor.test function). LncRNA-mRNA pairs that met the criteria of pearson correlation (|r| > 0.81) and adjusted p-value ≤ 0.05 were considered as putative cis interactions of lncRNAs (Babaei et al., 2024).

Protein-coding genes located more than 100 kb from the lncRNA locus were considered as potential trans targets. Trans target identification was performed on protein-coding genes using the same correlation framework (Pearson and Spearman correlation coefficients) and multiple-testing correction was applied across the tested trans-acting lncRNA-mRNA pairs using the Benjamini-Hochberg false discovery rate (FDR) method (Haynes, 2013). LncRNA-mRNA (Pearson correlation |r| > 0.81 and raw p-value ≤ 0.05) were retained as putative trans-acting associations. Trans interactions were validated using LncTar (Li et al., 2015), which estimates the normalized binding free energy (ndG) between co-expressed lncRNAs and their paired protein-coding genes. Given the non-canonical nature of lncRNA-mRNA interactions in plants, only lncRNA-mRNA interaction pairs with ndG ≤ -0.08 were retained as energetically plausible interactions, as reported previously (Li et al., 2015; Jali and Sharma, 2025).

### 2.8 Prediction of lncRNA-miRNA-mRNA regulatory network

To infer the functions of DE lncRNAs, a lncRNA-miRNA-mRNA regulatory network was constructed based on predicted RNA-RNA interactions. Mature miRNA sequences of A. thaliana were obtained from the miRBase database (Release 22.1) (Kozomara et al., 2019). The psRNATarget tool (Dai et al., 2018) was used to predict interactions between DE lncRNAs and miRNAs, as well as between miRNAs and DE protein-coding mRNAs, applying an expectation cut-off of ≤ 3.5 for miRNA-mRNA and ≤ 4.5 for lncRNA-miRNA interactions. The resulting interaction pairs were used to construct the regulatory network and were visualized using Cytoscape (Shannon et al., 2003).

### 2.9 Functional prediction and enrichment analysis of lncRNAs

Functional prediction of lncRNAs was based on their associated protein-coding target genes. The DE target mRNAs identified from cis- and trans-acting lncRNA associations and the lncRNA-miRNA-mRNA regulatory network were used for enrichment analysis. The Arabidopsis Araport11 genome annotation was used to construct the background gene set. Gene Ontology (GO) term analysis was conducted using the clusterProfiler package in R (Yu et al., 2012), with statistical significance assessed at an adjusted p-value (Padj ≤ 0.05). Additionally, the functional descriptions of the target genes were annotated using publicly available TAIR10 genome assembly (Araport11 gene annotation) resources, and a heatmap was generated in R using the Complex Heatmap package (Gu et al., 2016).

### 2.10 Computational environment and data processing

Data processing, statistical analysis, and result integration were performed in the R environment using RStudio. RNA-seq analysis, including read processing, was conducted on the Galaxy server (Blankenberg et al., 2014), while downstream handling, filtering, statistical testing, and visualization were done locally in R. Plots were generated with common libraries like ggplot and VennDiagram (Chen and Boutros, 2011), and schematic illustrations were created using BioRender. Custom Bash scripts were used for specific data preprocessing tasks via the command line. An overview of the RNA-seq workflow is in **Supplementary Table S7**, and the software tools used, along with their versions, settings, and computational platforms, are detailed in **Supplementary Table S8**. The codes used for the analysis in this study are provided in the GitHub repository (https://github.com/nischaypatel4/LncRNA-Analysis-Pollination-Arabidopsis).

## 3 Results

### 3.1 Sense genic lncRNA transcripts are predominant among expressed predicted novel lncRNAs

**Figure 1:**
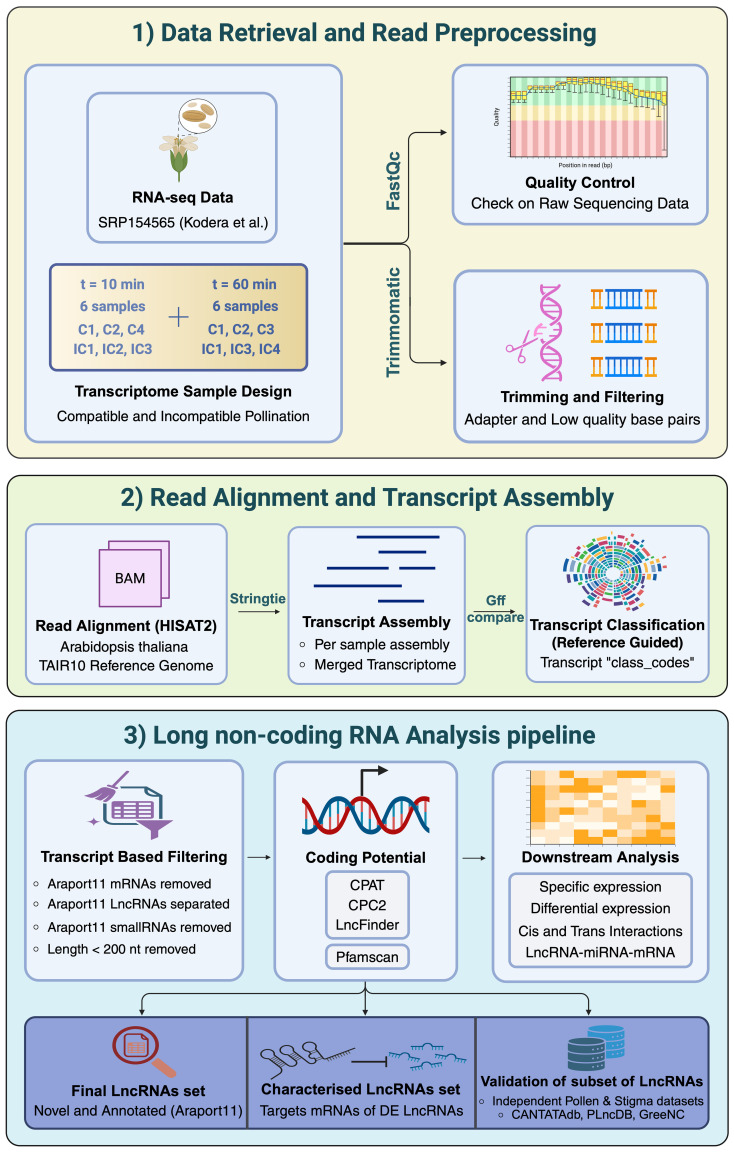
Overview of the RNA-seq data workflow for processing, identifying, characterizing, and analyzing long non-coding RNAs. TAIR10 genome assembly (Araport11 gene annotation) was used as a reference in this study. The figure was created using BioRender.com.

LncRNAs associated with compatible and incompatible pollination responses were determined from RNA-seq data of pollinated *A. thaliana* stigmatic tissues using the workflow illustrated in **Figure 1**. The dataset comprised a total of 12 RNA-seq samples of pollinated stigmatic tissues from compatible and incompatible pollination at early (t = 10 min) and late (t = 60 min) post-pollination stages, with three biological replicates per condition and time point. Only paired-end reads that passed the filtering criteria were retained for further analysis. The proportion of discarded read pairs ranged from 1.72% to 2.46% across the samples (**Supplementary Table S9**). The alignment of high-quality reads to the A. thaliana TAIR10 genome assembly (Araport11 gene annotation) using HISAT2 displayed alignment rates of more than 97% across the samples (**Supplementary Table S5**), indicating high-quality sequencing and mapping.

**Figure 2:**
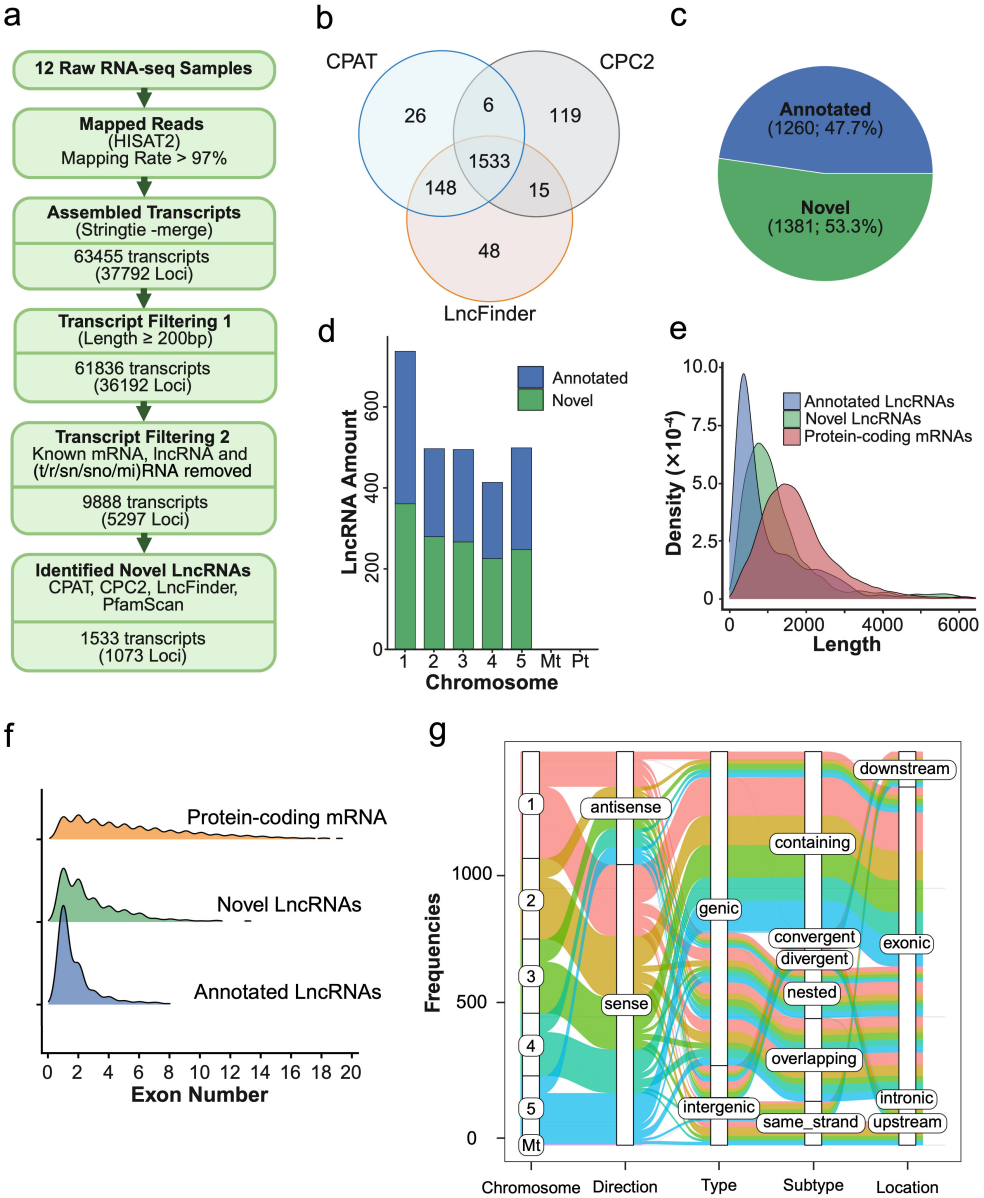
Characteristic features of annotated and predicted novel long non-coding RNAs identified in pollinated stigmatic tissues of compatible and incompatible pollination in *Arabidopsis thaliana* at early (t = 10 min) and late (t = 60 min) post-pollination. The image denote, **(a)** Schematic overview of the bioinformatic workflow used for RNA-seq processing, transcript assembly, sequential filtering, and identification of high-confidence predicted novel lncRNAs **(b)** Venn diagram showing the overlap of putative non-coding transcripts predicted by CPAT, CPC2, and LncFinder **(c)** Proportion and absolute numbers of annotated and novel lncRNA-producing loci among expressed lncRNAs **(d)** Chromosomal distribution of annotated and predicted novel lncRNA-producing loci **(e)** Length distribution of expressed annotated lncRNAs, expressed predicted novel lncRNAs, and expressed protein-coding mRNAs **(f)** Exon number distribution of expressed predicted novel lncRNAs, expressed annotated lncRNAs, and expressed protein-coding mRNAs **(g)** Genomic classification of expressed predicted novel lncRNAs based on positional relationship to neighboring protein-coding genes. Figure 2a was created using BioRender.com.

The merged transcriptome consisted of 63,455 predicted transcripts corresponding to 37,792 genomic loci (**Figure 2a**). After filtering for transcripts longer than 200 bp, 61,836 transcripts (36,192 loci) were retained. The removal of known protein-coding and annotated biotypes (tRNA, rRNA, snRNA, snoRNA, and miRNA) resulted in 9,888 potential candidate transcripts that were subjected to coding-potential assessment. Additionally, 3,836 transcripts (corresponding to 3,422 loci) that were annotated with the biotype long non-coding RNA (lncRNA) in Araport11 were identified and retained as annotated lncRNAs. These transcripts were excluded from the coding-potential assessment and were subsequently combined with the novel lncRNAs identified in this study to generate the final lncRNA dataset used for all downstream analysis. Coding potential assessment using three independent tools, CPAT, CPC2, and LncFinder, effectively identified 1,533 predicted novel lncRNA transcripts associated with 1,073 lncRNA loci (**Figure 2b**). None of these transcripts displayed specific functional or structural protein domains using the Pfam database (**Supplementary Table S10**). In total, 1,381 predicted novel lncRNA transcripts derived from 1,002 expressed lncRNA loci met the TPM and replicate consistency criteria. In addition, 1,260 of the 3,836 annotated lncRNA transcripts, representing 985 of the 3,422 annotated lncRNA loci, were expressed, resulting in a comprehensive dataset comprising both expressed annotated and novel lncRNAs (**Figure 2c**; **Supplementary Table S11**).

The expressed lncRNAs loci were unevenly distributed on chromosomes, with the highest number associated with chromosome 1 (**Figure 2d**). The predicted novel lncRNAs were generally shorter than annotated lncRNAs and protein-coding mRNAs, with mean transcript lengths of 1,186 bp for lncRNAs and 1,830 bp for protein-coding transcripts (**Figure 2e**). Consistent with known features, predicted lncRNAs contained fewer exons than protein-coding genes, while novel lncRNA transcripts exhibited a higher exon count compared with annotated lncRNA transcripts (**Figure 2f**). Detailed information on chromosomal distribution, transcript length, and exon number for both identified and expressed annotated and novel lncRNAs is provided in **Supplementary Tables S10**, **S11**. The structural annotation file (GTF) corresponding to the annotated and novel lncRNAs identified in the study is provided as **Supplementary Tables S12A, B**.

Genomic classification of the 1,381 expressed predicted novel lncRNA transcripts revealed that the majority of them were sense lncRNAs (70.4%, 972/1,381), while 29.6% (409/1,381) were antisense. Among the sense transcripts (n = 972), 83.5% (812/972) belonged to genic, and the remaining 16.5% (160/972) were intergenic. Similarly, the assessment of the antisense subset (n = 409) showed that 67.5% (276/409) were genic and 32.5% (133/409) were intergenic. Together, these results suggest that sense-genic lncRNAs were abundantly expressed among the novel lncRNAs (**Figure 2g**). The detailed FEELnc-based genomic classification of novel lncRNAs information and location (exonic, intronic, downstream and upstream) with respect to their related mRNA are provided in **Supplementary Table S13**.

### 3.2 Late post-pollination stage is characterized by an increased number of predicted DE lncRNAs

**Figure 3:**
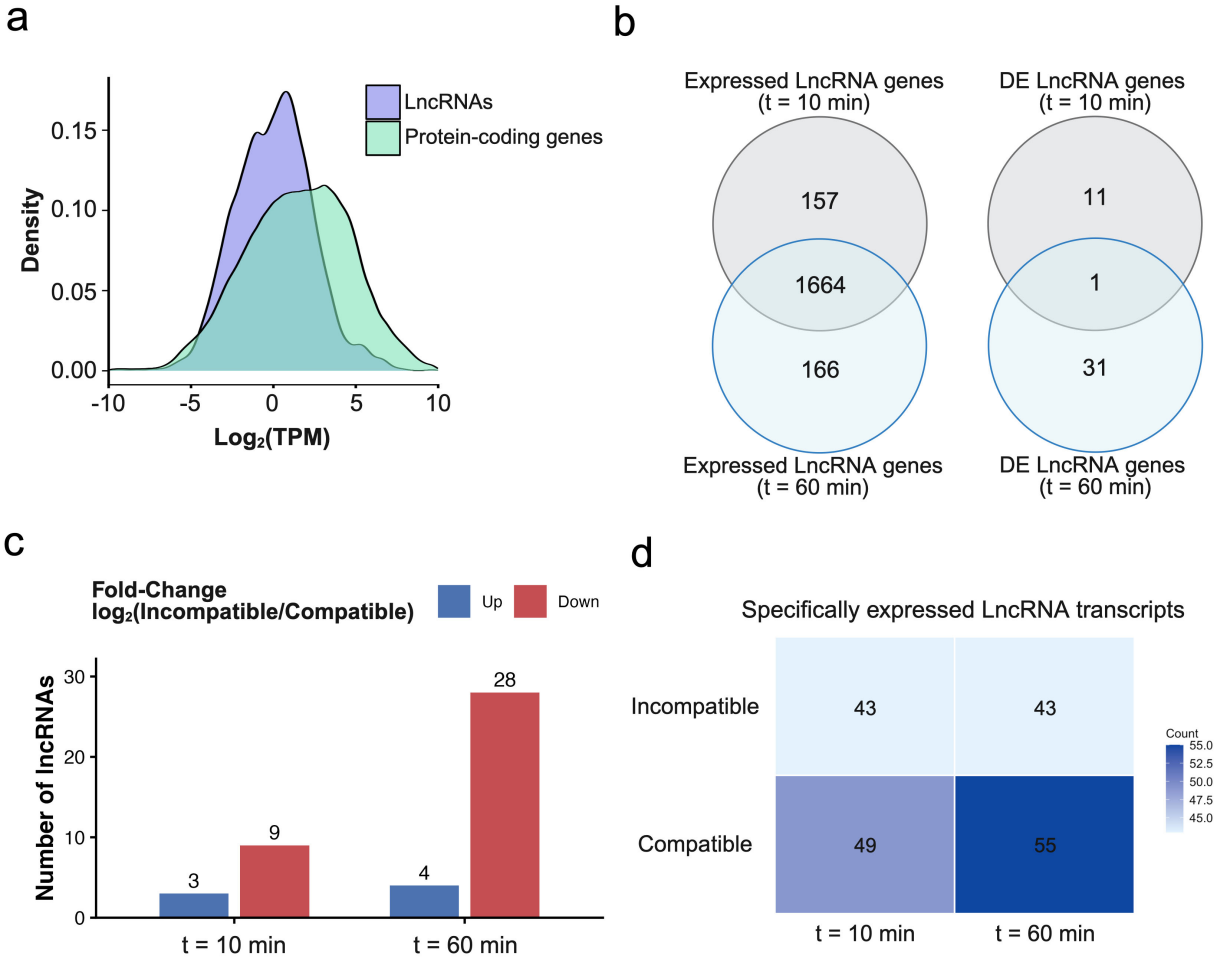
Expression classification and temporal expression pattern of predicted lncRNAs in pollinated stigmatic tissues of compatible and incompatible pollination in *Arabidopsis thaliana* at early (t = 10 min) and late (t = 60 min) post-pollination. The image displays **(a)** Density distribution of transcript expression levels showing log_2_-transformed TPM values for lncRNAs and protein-coding genes across all samples. LncRNAs exhibit lower overall expression levels than protein-coding genes. **(b)** Venn diagrams depicting the number of expressed lncRNA genes (left) and predicted differentially expressed (DE) lncRNA genes (right) shared between t = 10 min and t = 60 min, as well as those specific to each time point. Differential expression was assessed as incompatible vs compatible pollination conditions. **(c)** Number of upregulated and downregulated DE lncRNAs at t = 10 min and t = 60 min, based on fold change = log_2_(TPM Incompatible Sample)/(TPM Compatible Sample). **(d)** Heatmap summarizing specifically expressed lncRNA transcripts, defined as transcripts expressed in one pollination condition (compatible or incompatible) and absent in the other at the same time point.

LncRNAs exhibited lower expression levels than protein-coding genes, as reflected by the distribution of log_2_(TPM) values (**Figure 3a**) (Grammatikakis and Lal, 2022). Determination of the mean-variance relationship of transcript expression further revealed increased variability at low expression levels (TPM ≤ 0.05) (**Supplementary Figure S2**). Based on stringent criteria for TPM thresholds and replicate consistency, a total of 1,821 and 1,830 predicted lncRNA loci were identified as expressed at t = 10 min and t = 60 min, respectively. Among these, 1,664 loci were expressed at both time points, whereas 157 loci were uniquely expressed at t = 10 min and 166 loci were specific to t = 60 min, respectively (**Figure 3b**). Thus, the expressed number of lncRNA loci was comparable between early and late post-pollination stages (**Supplementary Table S11**).

DE analysis of incompatible and compatible pollination (standard significance threshold adjusted p-value < 0.05) identified 37 predicted DE lncRNA loci across both time points. A relatively relaxed significance threshold adjusted p-value < 0.1 resulted in 43 DE lncRNA loci, which were retained for the subsequent analysis. An absolute log2(TPM Incompatible Sample)/(TPM Compatible Sample) ≥ 1 was used for both standard and relaxed significance threshold. Only a single DE lncRNA locus (*XLOC_004982*) was shared between t = 10 min and t = 60 min, whereas 11 loci were specific to t = 10 min and 31 loci were specific to t = 60 min (**Figure 3b**). Thus, despite broadly similar numbers of expressed lncRNA loci at both time points, a substantially larger number of DE lncRNAs was detected at 60 min, highlighting a temporal shift in lncRNA regulation from t = 10 min to t = 60 min post-pollination stage. The majority of predicted DE lncRNAs were downregulated under incompatible pollination conditions. At t = 10 min, 9 out of 12 DE lncRNAs were downregulated, while at t = 60 min, 28 out of 32 DE lncRNAs showed downregulation. Overall, 36 (one common DE lncRNA *XLOC_004982* between t = 10 min and t = 60 min) of the 43 DE lncRNAs were downregulated under incompatible pollination (**Figure 3c**). The most significant downregulation of predicted DE lncRNAs was found at 10 min (fold change = -7.8), and a comparatively less downregulation at 60 min (-3.7). In contrast, mRNA showed a fold change of -8.5 at 10 min but strong upregulation of 9.8-fold at 60 min. The corresponding predicted DE lncRNAs and DE protein-coding mRNAs identified in this study are provided in **Supplementary Tables S14**, **S15**, while DESeq2-normalized expression values are given in **Supplementary Tables S16A, B**.

Finally, lncRNA transcripts showing condition-specific expression were defined as those expressed in only one pollination condition (compatible or incompatible) at a given time point (t =10 min or t = 60 min). A higher number of specifically expressed lncRNA transcripts was observed under compatible pollination conditions at both t = 10 min and t = 60 min (**Figure 3d**; **Supplementary Tables S17A, B**).

### 3.3 Predicted differentially expressed lncRNAs were expressed in independent stigma and pollen tissue datasets

The predicted DE lncRNAs during incompatible and compatible pollination across both time points validated against independent stigma and pollen datasets (using Arabidopsis RNA-seq Database) showed that most of these lncRNAs were either expressed in stigma or pollen tissues, furthering confirmation that these lncRNAs are specific to reproductive tissues. More specifically, *AT4G17098*, *AT5G49152*, and *AT1G07128* were expressed across independent pollen libraries, while *AT5G36002*, *AT1G31485*, *AT1G31935*, *AT1G07943*, *AT3G29644*, *AT3G51238* and *AT1G08043* were expressed in stigmatic tissue, particularly in stigmatic papillae across developmental time points (24 hr, 48 hr and 72 hr). Notably, *AT1G15405* (with maximum FPKM of 326.04 in pollen tissue) showed a consistent higher level of expression in both pollen and stigma tissues. The details for RNA-seq libraries for independent pollen and stigma tissues with average FPKM expression are provided in **Supplementary Table S18**.

Further verification of predicted DE and novel lncRNAs in PLncDB v2.0, CANTATAdb3.0, and GreeNC v2.0 databases showed that 488 out of 1,381 predicted expressed novel lncRNA transcripts (corresponding to 357/1,002 novel lncRNA loci) were shared with lncRNAs validated in these databases. Specifically, 20 DE lncRNA loci (9 annotated and 11 novel) out of the predicted 43 total DE lncRNA loci identified across both time points were also found in these databases. In addition, predicted 91 novel lncRNA transcripts (corresponding to 66 novel lncRNA loci) identified in our study matched with PLncDB v2.0 lncRNA catalogue consists of data generated by qRT-PCR or RT-PCR or microarray in *A. thaliana*. The details of the hits obtained for lncRNAs in these databases are provided in **Supplementary Table S19**.

### 3.4 Predicted lncRNAs showed a limited number of potential cis-acting mRNA targets across both time points

Cis interactions have been widely reported in plants, where lncRNAs modulate the expression of neighbouring genes through local transcriptional or chromatin-based mechanisms, enabling rapid and specific transcriptional responses during developmental and stress-related processes (Chekanova, 2015; Lucero et al., 2021).

A total of 15 potential cis-acting lncRNA-mRNA pairs were analyzed across the two post-pollination time points. Among these, 10 significant cis interactions were found, involving seven predicted DE lncRNAs and nine DE mRNAs. A higher number of potential cis interactions was observed at the 60 min. The identified cis interactions exhibited strong expression correlations (|r| = 0.869-0.997). Of the predicted ten significant cis pairs, eight interactions showed positive correlations, while two interactions showed negative correlations. Notably, seven out of ten predicted cis interactions exhibited very strong correlations (|r| > 0.90). Consistent with the direction of correlation, eight predicted cis pairs displayed concordant regulation (both lncRNA and mRNA either upregulated or downregulated), whereas two pairs exhibited discordant regulation, characterized by opposite expression trends between the lncRNA and its neighbouring mRNA.

The predicted cis-associated target mRNAs included genes such as *PCR2* (*AT1G14870*), *MAP3K13* (*AT1G07150*), and *DREB26* (*AT1G21910*), along with genes encoding pathogenesis-related thaumatin-like proteins, thionin-like peptides, chalcone-flavanone isomerase family proteins, phenolic glucoside malonyl transferase (PMAT1), SKU5-like proteins, and an ATPase F subunit. Among these, *PCR2* and a *thionin-like gene* were identified as predicted overlapping targets with their associated lncRNAs, whereas the remaining cis interactions involved neighbouring but non-overlapping genes. The predicted significant cis interactions, together with their genomic distances, correlation coefficients, statistical significance values, and functional annotations, are summarized in **Table 1**; **Supplementary Table S20**.

**Table 1:** Predicted significant cis-acting lncRNA-mRNA interaction pairs identified at early (t = 10 min) and late (t = 60 min) pollination time points.

Time stage	lncRNA locus (IC/C)	mRNA gene (IC/C)	Pearson correlation	Distance	Target description
t10	*XLOC_004982* (Down)	*AT1G14870* (Down)	0.965**	0	*PCR2* encodes a membrane protein involved in zinc transport and detoxification.
t10	*XLOC_029258* (Down)	*AT5G40020* (Up)	-0.894*	15611	Pathogenesis-related thaumatin superfamily protein;(source:Araport11)
t60	*AT1G07128* (Down)	*AT1G07150* (Up)	-0.932*	-5545	Member of MEKK subfamily. Involved in wound induced signaling where it interacts with At5g40440; and activates At1g59580.
t60	*AT3G51238* (Down)	*AT3G51230* (Down)	0.961**	758	chalcone-flavanone isomerase family protein;(source:Araport11)
t60	*XLOC_001211* (Down)	*AT1G21860* (Down)	0.923*	-1287	SKU5 similar 7;(source:Araport11)
		*AT1G21866* (Down)	0.897*	0	Thionin-like gene.
		*AT1G21910* (Down)	0.869*	18397	encodes a member of the DREB subfamily A-5 of ERF/AP2 transcription factor family. The protein contains one AP2 domain. There are 15 members in this subfamily including RAP2.1; RAP2.9 and RAP2.10.
t60	*XLOC_004982* (Down)	*AT1G14870* (Down)	0.973**	0	*PCR2* encodes a membrane protein involved in zinc transport and detoxification.
t60	*XLOC_029189* (Up)	*AT5G39050* (Up)	0.997**	-2017	Encodes a malonyl transferase that may play a role in phenolic xenobiotic detoxification.
t60	*XLOC_031012* (Down)	*ATCG00130* (Down)	0.921*	2064	ATPase F subunit.

The table presents predicted differentially expressed lncRNAs and their associated cis-target protein-coding genes, including the direction of expression change (up/down), Pearson correlation coefficients, genomic distances between lncRNA and target gene loci, and functional annotations of the target genes. Functional annotations were obtained from Araport11. Asterisks in the “Pearson correlation” column indicate significance levels (*p < 0.05; **p < 0.01). A comprehensive list of significant cis interactions is provided in **Supplementary Table S20**.

### 3.5 Predicted trans-regulatory associations of lncRNAs are enhanced during the late post-pollination stage

**Figure 4:**
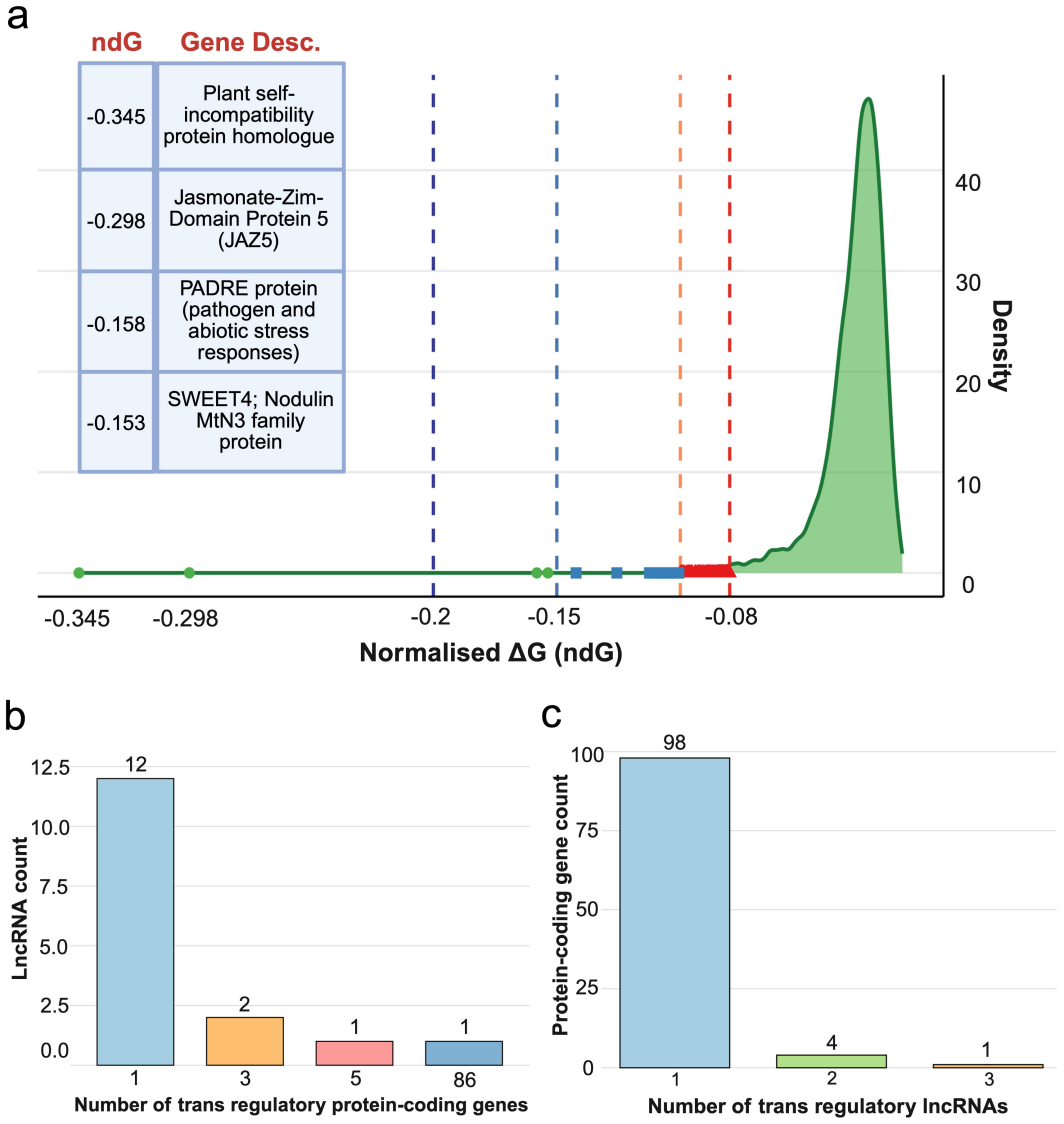
Predicted lncRNA-mRNA trans interaction landscape. The image shows **(a)** Density distribution of normalized binding free energy (ndG) values for predicted lncRNA-mRNA trans interactions after filtering. The red dashed line indicates the ndG cutoff (≤ -0.08). Interactions are categorized by ndG ranges: red triangles (-0.08 to -0.10), blue squares (-0.10 to -0.15), and green circles (≤ -0.15). The predicted four targets with the most negative ndG values are highlighted. **(b)** Distribution of lncRNAs based on the number of predicted trans-regulatory protein-coding target genes. **(c)** Distribution of protein-coding genes according to the number of trans-regulatory lncRNAs.

The trans interaction landscape of predicted DE lncRNAs was assessed using the distribution of ndG values for predicted lncRNA-mRNA pairs using a density plot (**Figure 4a**). The trans interactions displayed a notable skew toward the right side of the chosen cutoff, indicating a high density of energetically permissible but relatively weaker interactions observed at ndG values 0.08 (ndG ≥ -0.08). A significant decline in interaction density was observed around the ndG = -0.08 threshold. Most of the predicted interactions fell within the range (-0.08, -0.1), and the number of interactions declined as the predicted interaction strength increased. A total of predicted 109 significant trans-acting lncRNA-mRNA associations, consisting of 16 DE lncRNAs and 103 protein-coding genes, were found using combined expression correlation (|r| ≥ 0.81) and structural filtering (ndG ≤ -0.08). Of these, 107 predicted interactions were specific to the t =60 min, whereas only two interactions were detected at t = 10 min, indicating a strong enrichment of trans regulatory interactions at the later stage of pollination (**Supplementary Table S21**).

To further investigate the regulatory network architecture of lncRNA-mediated trans regulation, the distribution of predicted trans target genes per lncRNA was analyzed (**Figure 4b**). The majority of lncRNAs were associated with a single trans target, indicating that most lncRNAs exhibit limited trans-regulatory potential. Fewer lncRNAs targeted two or three genes, and only a very small subset exhibited higher target counts. Interestingly, four lncRNAs displayed the majority of predicted trans interactions, where the lncRNA *AT3G04795* was associated with 86 predicted protein-coding trans targets, while *XLOC_017394*, *XLOC_001211*, and *XLOC_020056* were linked to five, three, and three targets, respectively. These four multi-target lncRNAs accounted for approximately 89% of all predicted trans interactions, suggesting that trans regulatory interactions were dominated by a few highly connected lncRNAs. The predicted targets for *AT3G04795* included genes annotated as members of the plant self-incompatibility protein S1 family, the PADRE protein family, and the plant thionin protein family (Araport11). Of the 103 unique protein-coding genes identified as trans targets, 98 were associated with only a single lncRNA, indicating that most coding genes are regulated by a unique lncRNA in trans. In contrast, only five protein-coding genes were targeted by more than one lncRNA (**Figure 4c**), indicating that convergent trans regulation by multiple DE lncRNAs was very limited.

Four target genes, including plant self-incompatibility protein homologs, *JAZ5*, *PADRE*, and *SWEET4*, exhibited the most negative ndG values among the predicted trans interactions (**Figure 4a**), indicating the strongest predicted lncRNA-mRNA binding energies. These interactions fall well beyond the primary ndG cutoff (≤ -0.08) and reside in the extreme left tail of the ndG distribution, representing a small subset of energetically highly favourable RNA-RNA hybridization events. In addition to these, several other high-confidence targets with strong energetic support, including RALF-like peptides (RALFL12 and RALFL13), the exocyst subunit EXO70, cell wall-modifying enzymes (CSLC4 and XTR6), the transcription factor MYB97, and the membrane-associated protein MLO12, were also predicted. The ten selected trans-target genes are summarized in **Table 2**.

**Table 2:** Selected trans-acting predicted lncRNA-mRNA interaction pairs at t = 60 min (t60).

Time stage	lncRNA locus (IC/C)	mRNA Gene (IC/C)	ndG	Target description	Relevance
t60	*AT3G04795* (Down)	*AT3G26880* (Down)	-0.3435**	Plant self-incompatibility protein S1 family	Pollen-stigma recognition; compatibility signaling
		*AT1G07725* (Down)	-0.1039**	EXO70 family exocyst subunit	Vesicle trafficking during pollen tube growth
		*AT3G28180* (Down)	-0.1034*	Cellulose synthase-like protein	Cell wall biosynthesis/remodelling
		*AT4G25810* (Down)	-0.0939*	XTR6 (xyloglucan endotransglycosylase)	Cell wall loosening for pollen tube penetration
		*AT5G26090* (Down)	-0.0880**	Plant self-incompatibility protein S1 family	Reinforces recognition-related compatibility pathways
t60	*XLOC_010850* (Up)	*AT3G28007* (Up)	-0.1536*	SWEET4 sugar transporter	Carbohydrate supply to compatible pollen
t60	*XLOC_028909* (Down)	*AT2G19040* (Down)	-0.1048**	RALF-like peptide (RALFL12)	Direct regulator of pollen tube growth
t60	*XLOC_026728* (Down)	*AT2G19045* (Down)	-0.0876**	RALF-like peptide (RALFL13)	Fine-tuning of pollen tube elongation
t60	*XLOC_017394* (Down)	*AT4G26930* (Down)	-0.0827**	MYB97 transcription factor	Pollen tube-synergid communication
t60	*AT3G04795* (Down)	*AT2G39200* (Up)	-0.0813*	MLO family protein (MLO12)	Membrane-level signaling in stigma/anther tissues

** indicates Pearson correlation > 0.9 with significant raw p-value < 0.01. * indicates Pearson correlation > 0.85 with significant raw p-value < 0.05 between predicted DE lncRNA and DE mRNA.

### 3.6 Late post-pollination displayed expanded network of predicted lncRNAs, miRNAs, and mRNA targets

LncRNAs can regulate gene expression by interacting with microRNAs (miRNAs) and protein-coding genes, and lncRNA-miRNA-mRNA regulatory networks are mostly formed by competing for shared miRNAs or modulating miRNA-mediated repression (Cesana et al., 2011; Salmena et al., 2011). To investigate this possible regulatory mechanism during pollination responses, we constructed lncRNA-miRNA-mRNA networks by integrating DE lncRNAs, mRNAs, and known miRNAs from the miRbase database. We first mapped possible mRNA-miRNA interactions and then lncRNA-miRNA interactions. The analysis of the integrated lncRNA-miRNA-mRNA regulatory network predicted 31 lncRNAs, 55 miRNAs, and 144 unique protein-coding genes across both time points. The early time point (10 min) showed only five lncRNAs, seven miRNAs, and 19 mRNA targets, whereas the late time point (60 min) displayed a substantially expanded predicted network with 26 lncRNAs, 51 miRNAs, and 128 mRNA targets (**Supplementary Table S22**).

Putative miRNA sponge interactions consist of pairs of lncRNA, miRNA, and mRNA, where lncRNA and their corresponding mRNA target show a consistent pattern of expression, either upregulation or downregulation for both, whereas opposing regulatory interactions are those where opposite expression changes between lncRNAs and their target mRNAs are found (Babaei et al., 2024). Using this criterion, we predicted 28 lncRNAs that can function as potential miRNA sponges, interacting with 110 unique protein-coding genes through 49 miRNAs. This consistent pattern of expression suggests that certain lncRNAs may regulate target gene expression by sequestering shared miRNAs and thereby alleviating miRNA-mediated repression during pollination responses.

**Figure 5:**
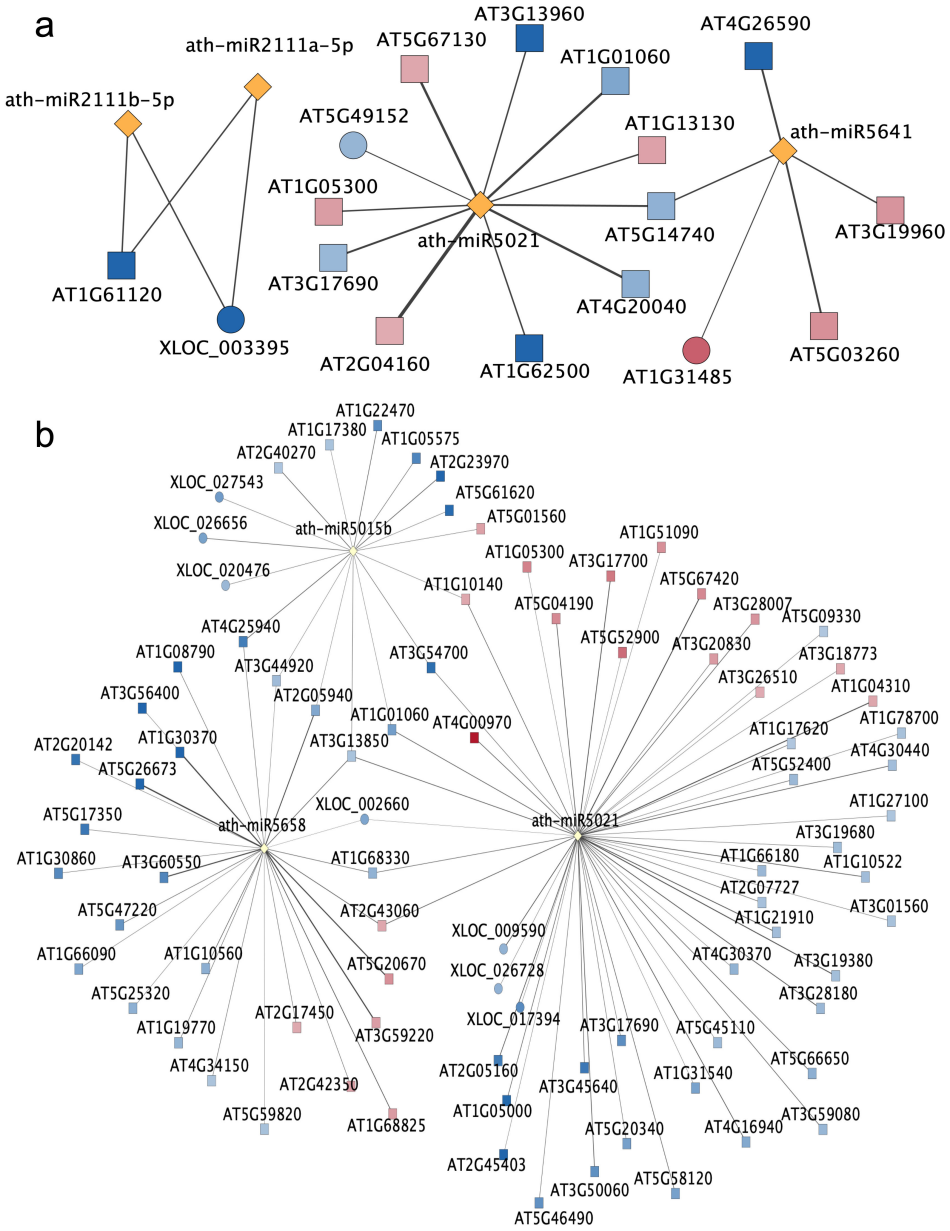
Predicted lncRNA-miRNA-mRNA interaction networks. The image presents **(a)** Predicted lncRNA-miRNA-mRNA regulatory network at 10 min post-pollination. **(b)** Predicted LncRNA-miRNA-mRNA regulatory network at 60 min post-pollination. Only miRNAs exhibiting high-confidence interactions with multiple lncRNAs and mRNA targets were included to ensure clarity of network visualization. Circles represent lncRNAs, rectangles represent mRNA targets, and yellow diamonds represent miRNAs. Node color indicates relative expression changes between incompatible and compatible pollination (IC/C), with increasing blue intensity denoting down-regulation and increasing red intensity denoting up-regulation.

Among the predicted miRNAs, ath-miR5021 and ath-miR5015b were the two most prominent miRNA hubs across both time points (**Figures 5a, b**). ath-miR5021 was associated with predicted DE 54 unique protein-coding target genes, of which 17 were upregulated and 37 were downregulated, while ath-miR5015b regulated 17 unique targets, including four upregulated and 13 downregulated genes. These hub miRNAs interacted with multiple predicted lncRNAs and target genes, highlighting their key role in shaping lncRNA-miRNA-mRNA regulatory networks during both early and late pollination responses. The predicted key miRNA hubs targeted genes involved in defense and stress responses, including *NPR3*, *PUB25*, and *TIR-NBS-LRR* immune receptors, as well as regulators of hypoxia and hormone signaling, such as *HUP17*, *Carbonic Anhydrase* (*CA2*), *JAZ5*, and *ERS2*. In addition, targets involved in pollen-pistil interaction-related processes, including *SWEET4*, *GAE1*, and *β-1,3-glucanase* (*BG5*), were also predicted. The underlying edge and node attributes of lncRNA-miRNA-mRNA network used for the construction and visualisation of **Figure 5** is provided in **Supplementary Tables S23A, B**.

### 3.7 Heatmap displayed progressive increase in target gene expression from early to late post-pollination

**Figure 6:**
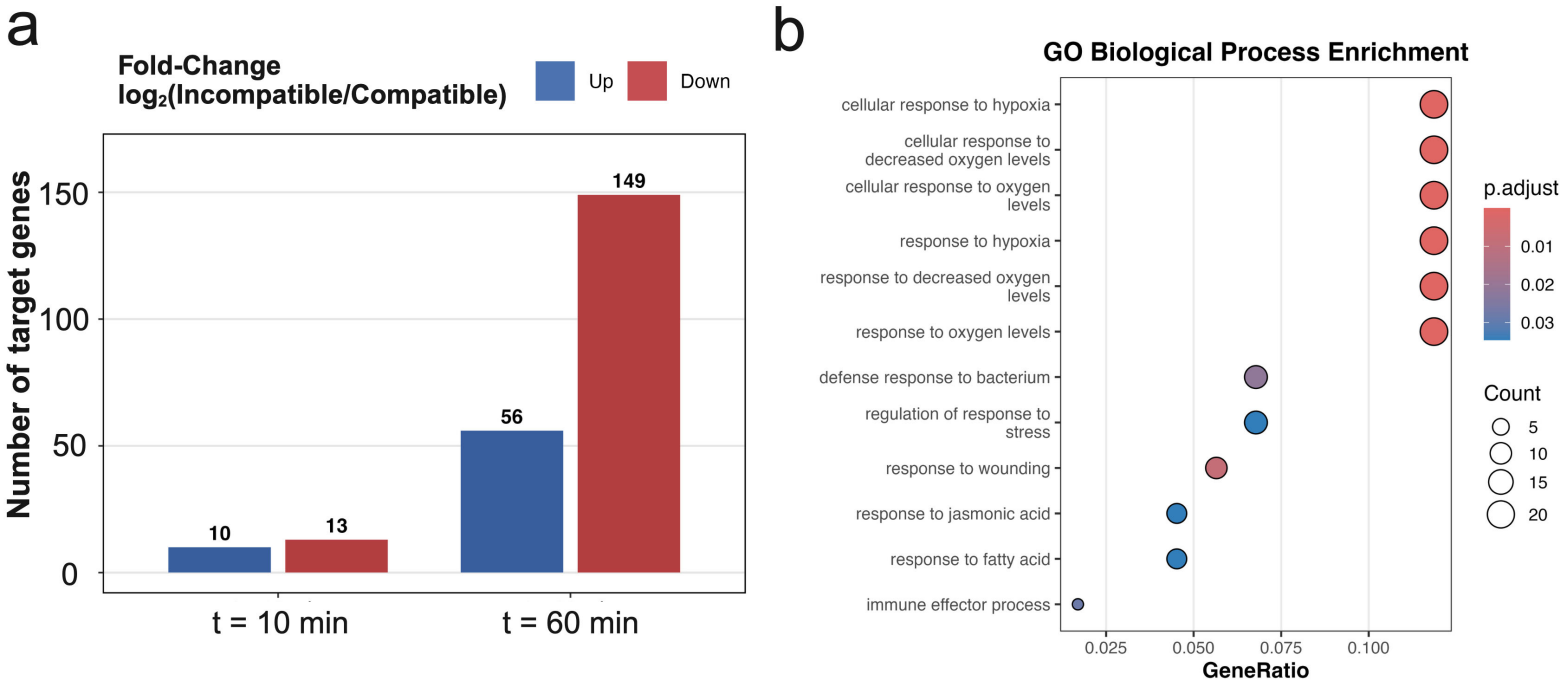
Temporal dynamics and functional enrichment of predicted differentially regulated target genes during compatible and incompatible pollination of *Arabidopsis thaliana.* The image shows **(a)** The number of predicted differentially expressed (DE) target genes at 10 and 60 min post-pollination, based on fold change = log_2_(TPM Incompatible Sample)/(TPM Compatible Sample). **(b)** Gene Ontology (GO) biological process enrichment analysis of the target genes, visualized as a dot plot. Dot size represents the number of genes associated with each GO term, while color intensity indicates the adjusted p-value. The x-axis denotes the gene ratio for each enriched biological process.

Post-pollination treatments at 60 min showed higher numbers of expressed predicted lncRNAs, DE lncRNAs, DE mRNAs, and associated target genes than at 10 min. The analysis of cis, trans, and lncRNA-miRNA-mediated possible interactions showed a notable increase in the number of differentially regulated target genes at 60 min compared to 10 min (**Figure 6a**). Gene Ontology (GO) enrichment analysis was performed using predicted target genes from interaction pairs between DE lncRNAs and DE mRNAs. The enriched GO terms included hypoxia response, stress regulation, defense mechanisms, and hormone signaling (**Figure 6b**; **Supplementary Table S24**).

**Figure 7:**
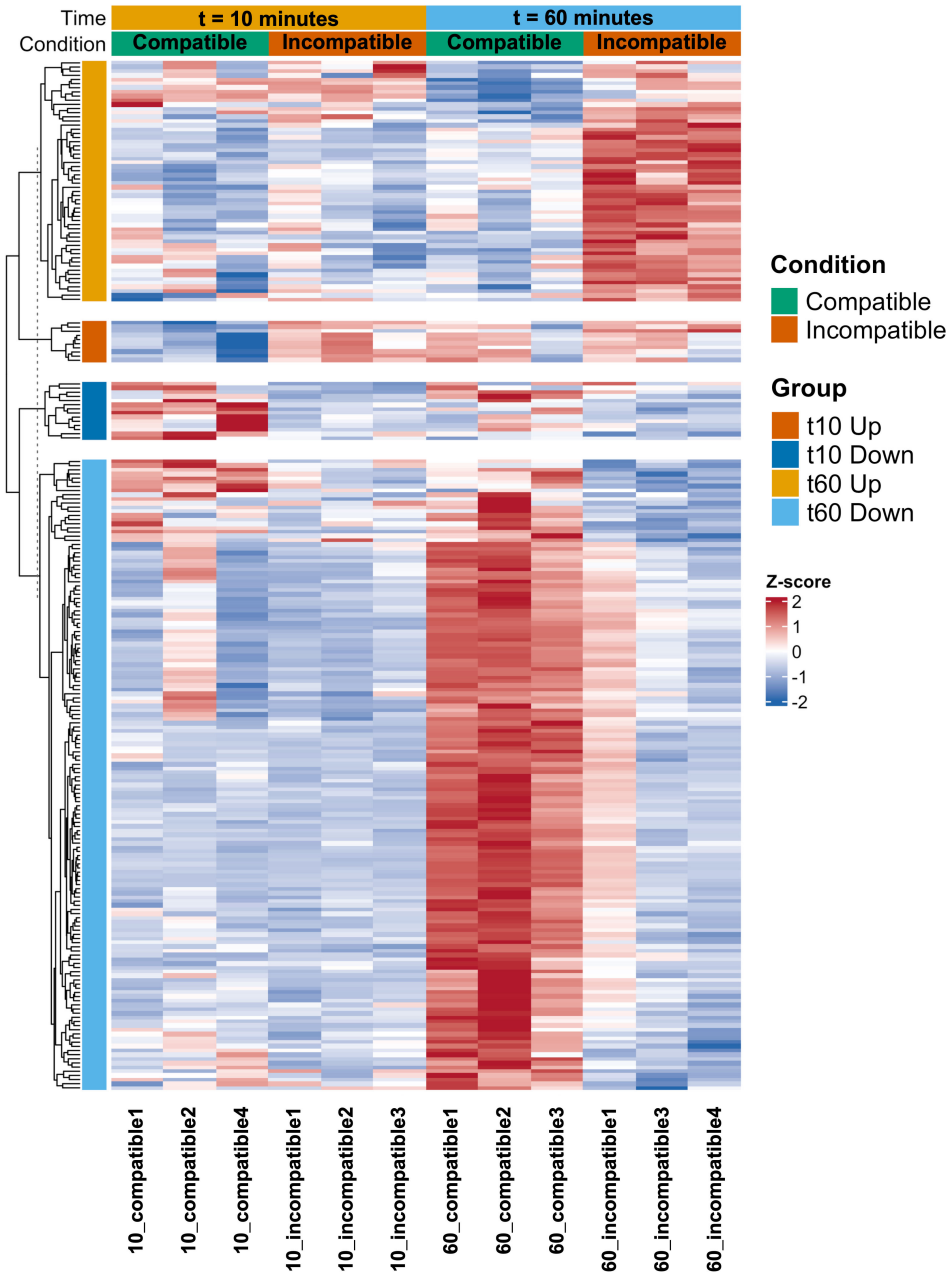
Global expression patterns of predicted target genes across time points and pollination conditions. Heatmap showing gene-wise Z-score scaled variance-stabilized expression of predicted target genes derived from cis-, trans-, and miRNA-mediated regulatory interactions. The heatmap includes all 12 samples representing compatible and incompatible pollination conditions at 10 and 60 min post-pollination. Genes are grouped into four sections based on their direction of regulation and the time point at which each predicted target was identified: targets upregulated at 60 min (t60 Up), targets upregulated at 10 min (t10 Up), targets downregulated at 10 min (t10 Down), and targets downregulated at 60 min (t60 Down), as determined by fold change (log_2_(TPM Incompatible Sample)/(TPM Compatible Sample) between incompatible and compatible conditions. Column annotations indicate time point and pollination condition, while colors represent relative expression levels, with red indicating higher and blue indicating lower expression relative to the gene-wise mean.

The global expression changes in lncRNA-associated target genes were visualized using a heatmap across both time points and pollination conditions (**Figure 7**), which included possible targets identified through cis, trans, and miRNA-mediated regulatory interactions across 12 samples. The target genes were categorized into four distinct groups based on the time point at which they were identified and their expression levels under pollination conditions. These categories included targets upregulated at 10 minutes (t10 Up), downregulated at 10 minutes (t10 Down), upregulated at 60 minutes (t60 Up), and downregulated at 60 minutes (t60 Down) (**Supplementary Table S25**). The targets predicted at 60 min showed more distinct and coordinated expression changes, particularly under incompatible conditions, consistent with the increased number of differentially regulated targets observed at 60 min (**Figure 7**). Together, this heatmap highlights the temporal specificity and progressive reprogramming of target gene expression during the transition from early to late pollination responses.

## 4 Discussion

During self-incompatibility responses in *Brassicaceae*, a cascade of signaling events is triggered. The stigma localized S-locus receptor kinase (SRK) recognizes the S-locus cysteine-rich protein (SCR/SP11) from self pollen, activating the downstream E3 ubiquitin ligase ARC1, leading to degradation of compatibility factors (Abhinandan et al., 2022, 2023; Bhalla et al., 2025a). In parallel, self-pollination induces ROS production through activation of FERONIA-mediated signaling, which disrupts pollen hydration and tube growth, resulting in rejection of self-pollen. In contrast, during compatible pollination, pollen coat proteins (PCP-Bs) suppress ROS production by competing with RALF23/33 peptides for interaction with the ANJEA-FERONIA receptor complex, thereby promoting pollen acceptance (Abhinandan et al., 2022; Bhalla et al., 2025b). To investigate the regulatory role of lncRNA in these pathways and identify novel molecular interactors, we performed an integrative analysis of lncRNAs, their mRNA targets, and associated miRNA networks using RNA-seq data. The datasets used in this study consisted of three replicates each for compatible and incompatible pollination at two time points, 10 min and 60 min post-pollination (Kodera et al., 2021). Since the stigmatic tissues datasets used in this study were pollinated with either compatible or incompatible pollen, the RNA-seq data capture transcriptional responses from the stigma and from the pollen as it grows through the stigmatic tissue. Before removing the replicates, the DESeq2 sensitivity analysis with and without excluded replicates for both 10 min and 60 min was performed. At the 60 min, with 4 replicates, the number of DE lncRNAs increased from 32 to 53, and the number of DE mRNAs increased from 502 to 743 (**Supplementary Table S26**). In contrast, at 10 min, when we used 4 replicates instead of 3 for the compatible and incompatible conditions, we found that the number of DE lncRNAs and DE mRNAs was drastically reduced (lncRNAs from 12 to 1; mRNAs from 129 to 2 (**Supplementary Table S26**). In addition, PCA plot analysis showed that 10_compatible3 and 10_incompatible4 did not cluster with the other three compatible or incompatible replicates at t = 10 min. So, we just excluded the most divergent replicates at each time point for each condition using a PCA plot and the ratio of mean to median distance based metric. To maintain consistency in the number of replicates across time points, we also used three replicates for each 60 min time point. A stringent three-step filtering approach using CPC2, CPAT, and LncFinder identified 9,888 lncRNA candidates, including 1,533 novel lncRNA transcripts spanning 1,073 lncRNA loci. This approach minimized false positives and enhanced dataset reliability.

The predominance of sense-genic lncRNAs suggests their potential involvement in regulating nearby protein-coding genes associated with pollination responses. LncRNAs were unevenly distributed across chromosomes; likely due to evolutionary selection pressures or to their functional specialization. However, the unique properties of lncRNAs may arise due to their distinct roles within the transcriptomic landscape. These findings highlight lncRNA diversity in reproductive processes and provide a foundation for future investigations into their regulatory mechanisms in gene expression. Their distinct expression patterns between compatible and incompatible pollination further support the notion that lncRNAs act as active regulators rather than byproducts of transcription.

Differential expression analysis of predicted lncRNA loci revealed that at 10 min, only 11 loci were expressed, whereas at 60 min, 31 loci were expressed, among which the majority of differentially expressed lncRNAs were downregulated in incompatible pollination, with the highest reduction of 7.8-fold (**Figure 3c**). These results suggest that later stages of pollination are associated with the activation of additional lncRNAs, which may have a significant regulatory role in reproductive success. The presence of a higher number of expressed lncRNA transcripts under compatible pollination at both t = 10 min and t = 60 min post-pollination suggests a potential role for these lncRNAs in promoting compatibility and inhibiting responses that might lead to pollen rejection. A similar trend of downregulation like DE lncRNAs was also observed in analysed DE mRNAs at similar time points in this study, suggesting the role of lncRNAs in fine tuning mRNA expression levels.

The validation of predicted DE lncRNAs during incompatible and compatible pollination with independent stigma and pollen datasets using (https://plantrnadb.com/athrdb/) confirmed 12 out of 43 predicted DE lncRNAs, which were expressed in pollen or stigma or both the tissues, suggesting that these lncRNAs were specific to reproductive tissues (**Supplementary Table S18**). To further confirm the other DE and predicted novel lncRNAs, we utilized databases that included PLncDB v2.0 (13,599 *A. thaliana* lncRNA transcripts compiled from experimentally supported records, RNA-seq datasets and public repositories, including RNAcentral and EVLncRNAs), CANTATAdb 3.0 (6,775 *A. thaliana* lncRNAs identified from high-throughput RNA-seq analysis), and GreeNC v2.0 (2,254 *A. thaliana* lncRNAs annotated using coding-potential-based filtering). These databases consist of lncRNAs that are expressed during various plant developmental stages and stress conditions, strengthening the reliability of our lncRNAs in *A. thaliana* (**Supplementary Table S19**).

The analysis of significant cis-acting predicted lncRNA-mRNA interaction pairs at t = 10 min and t = 60 min of post-pollination showed that, at both time points, the lncRNA *XLOC_004982* showed a strong positive correlation with the mRNA *AT1G14870* (*PCR2*), both of which were downregulated. *PCR2* belongs to the Arabidopsis PCRs, a small gene family of 12 members, including *PCR11*, which is expressed in pollen and belongs to the same clade as PCR2 (Song et al., 2010). *PCR2* encodes a protein involved in zinc transport and detoxification, suggesting that this gene may be a possible regulator for *XLOC_004982* in metal ion homeostasis during early and late post-pollination. Since, the RNA-seq data used in this study is generated from pollinated stigmas consisting of mixed tissues from stigma, pollen grains, and growing pollen tubes, the observed expression patterns for lncRNAs cannot be assigned to a specific cell type.

The analysis of differentially expressed genes (DEGs) in the pistil transcriptomes of *Arabidopsis thaliana* and *Arabidopsis halleri* during self-pollination, interspecific pollination, and pathogen infection with *Fusarium graminearum* showed that up to 79% of down-regulated genes were shared between pollination and pathogen response (Mondragón-Palomino et al., 2017). Meanwhile, interspecific pollination of *A. thaliana* upregulates thionins and defensins, which are involved in the defense mechanism. Consistent with this, our analysis identified several stress- and defense-related genes among lncRNA targets, including members of the MEKK subfamily (*AT1G07150*), which are implicated in wound-induced signaling, indicating a connection between physical stress responses and pollination. Downregulation of genes such as *Chalcone-flavanone isomerase* (*AT3G51230*) and DREB subfamily member *A-5* (*AT1G21910*) proteins may suggest a strategic shift toward prioritizing reproductive processes over stress responses. The negative correlation between *XLOC_029258* and *AT5G40020* at the early time point suggests a complex relationship in which the lncRNA may influence the expression of a pathogenesis-related protein. Similarly, the negative correlation between *AT1G07128* and *AT1G07150*, and the strong correlation between *XLOC_029189* and *AT5G39050* at 60 min. The cross-validation of *AT1G07128* lncRNA using independent reproductive tissue datasets showed its enrichment in pollen tissues, further suggesting its possible involvement in pollen-related reproductive processes compared to other tissues, such as the stigma and growing pollen tubes, during pollination, which needs further functional validation. The interaction between pollination and pathogen response indicates the complex relationship between plant reproductive strategies and defense mechanisms. This emphasizes the potential role of lncRNAs as key regulators of gene expression through significant lncRNA-mRNA interactions.

Predicted trans-acting lncRNA-mRNA interaction analysis identified key players involved in pollination. Most of these targets were predicted at 60 min post-pollination. The previously annotated lncRNA *AT3G04795* accounted for 86 out of 103 trans targets, and dominated the extended network. The members of the exocyst complex regulate polarized secretion and are important for the acceptance of compatible pollen in both Brassica and Arabidopsis (Samuel et al., 2009; Safavian et al., 2015). Among these members, EXO70A1, EXO70A2 and EXO70C2 are essential for pollen maturation, germination, and tube growth (Samuel et al., 2009; Marković et al., 2020; Saccomanno et al., 2021). Our results predicted the interaction between previously annotated lncRNA *AT3G04795* and EXO70 family exocyst subunit (AT1G07725), suggesting a possible role of these interactions in vesicle trafficking required for pollen tube growth. The downregulation of the previously annotated lncRNA *AT3G04795* is linked to several mRNA targets, including *AT3G26880* and *AT5G26090*, which encode a self-incompatibility protein S1 (SPH family member). The SPH family was initially identified in the self-incompatibility response of the field poppy (Rajasekar et al., 2019). *AT3G26880* and *AT5G26090* are highly expressed in the pollen tube of Col-0 (https://evorepro.sbs.ntu.edu.sg/), suggesting that lncRNA *AT3G04795* may negatively regulate the expression of these genes, possibly disrupting the pollen tube growth during incompatible pollination. The lncRNA *XLOC_010850* was predicted to interact with *AT3G28007* (*SWEET4*). The SWEET4 homolog in Arabidopsis, SWEET5, functions in later stages of pollen development, whereas SWEET4 is involved in sugar transport to axial tissues during plant growth and development (Liu et al., 2016). This possible interaction suggests that lncRNA may be involved in nutrient allocation during pollen development. RALF family members (e.g., RALF4/9) and their pollen-tube receptors Buddha’s Paper Seal 1 and 2 (BUPS1/2) are essential for normal pollen tube growth (Ge et al., 2017). *RALFL12* and *RALFL13* displayed higher expression in Col-0 pollen tubes (https://evorepro.sbs.ntu.edu.sg/). In our study, *XLOC_028909* and *XLOC_026728* (both downregulated) target *RALFL12/13*, suggesting their possible role during compatible pollination. However, the mixed tissue data for pollinated stigma used in our study does not allow confirmation of the cell or tissue-specific expression of these lncRNAs.

The MLO family proteins (MLO1, 5, 9, and 15) act as Ca^2+^ channels, enabling influx to sustain pollen tube integrity. RALF peptides bind pollen-tube receptors to activate these channels and establish a Ca^2+^ gradient (Gao et al., 2023). Our study shows that downregulation of annotated lncRNA (*AT3G04795*) coincides with upregulation of target *MLO12*, implying possible negative regulation. Previous studies have reported a shared pathway for the pathogen response and pollination (Kessler et al., 2010; Mondragón-Palomino et al., 2017). Mutations in *MLO* gene family members, especially *MLO2*, *MLO6*, and *MLO12*, limit powdery mildew colonization and influence interactions with a range of other phytopathogens. In our study, the interaction between annotated lncRNA *AT3G04795* and *MLO12* supports an integrated regulatory framework connecting pollination and disease resistance, with *MLO12* serving as a key component in this defense pathway and possibly involved in maintaining pollen tube integrity, as do other MLOs. MYB transcription factors regulate male reproductive development in flowering plants. In Arabidopsis, loss of pollen-specific *MYB97*, *MYB101*, and *MYB120* reduces the expression of pollen-tube-expressed genes and disrupts pollen ability to burst upon reaching a synergid cell, ultimately impairing fertilization (Leydon et al., 2014). These MYB genes are localized to the vegetative cell nucleus of the mature pollen grain or pollen tubes and are involved in coordinating pollen tube gene expression during pistil growth. In our study, lncRNA *XLOC_017394* targets *MYB97* (*AT4G26930*), and both these transcripts are downregulated, suggesting that lncRNA *XLOC_017394* may be a possible regulator *MYB97* expression, affecting pollen tube development and male reproductive success during the late post-pollination. However, these claims warrant further cell or individual reproductive tissue specific expression analysis and functional validation.

Among the predicted trans interactions, four target genes, including plant self-incompatibility protein homologs, *JAZ5*, *PADRE*, and *SWEET4*, exhibited the most negative ndG values (**Figure 4a**). The jasmonate hormone (JA) plays a critical role in both plant defense and reproductive development. In Arabidopsis, plants deficient in JA-biosynthesis or signaling are male-sterile, displaying defects in stamen and pollen development. In carrot protoplasts, the bHLH transcription factor MYC5 activates GUS reporter genes driven by the JAZ5 promoter, and MYC5 likely acts together with other transcription factors to induce *MYB21* and other players required for male fertility (Figueroa and Browse, 2015).

However, a limitation of the lncRNA-mRNA correlation analysis is that the correlation coefficients (pearson and spearman) were calculated using n = 6, comprising three replicates each from incompatible and compatible pollination at each timepoint (10 min and 60 min). To avoid the potential errors we excluded the zero or low expression values using the criteria at least four out of six samples should have TPM > 0.05 for both lncRNAs and mRNAs. Further, we calculated both pearson as well as spearman correlation coefficients and found that the lncRNA-mRNA pairs that showed pearson correlation coefficient |r| > 0.81 satisfy the criteria of spearman correlation coefficient of |ρ| > 0.75, except for pairs that included, *AT3G04795*-*AT3G51230* (|r| = 0.86, |ρ| = 0.71), *AT3G04795*-*AT4G22530* (|r| = -0.82, |ρ| = -0.65), *XLOC_020056*-*ATCG00350* (|r| = -0.83, |ρ| = -0.71), and *XLOC_031012*-*ATCG00130* (|r| = 0.92, |ρ| = 0.71).

LncRNAs are long, diverse regulatory RNAs transcribed by RNA polymerase II and are processed like mRNAs (capping, splicing, and polyadenylation). In contrast, miRNAs are short (~21 nt) RNAs derived from primary transcripts (pri-miRNAs) that are processed in plants by Dicer-like 1 (DCL1) into mature miRNAs, which are then introduced into the RNA-induced silencing complex (RISC) to guide sequence-specific binding to target mRNAs, resulting in mRNA cleavage (Yu et al., 2026). In our study, ath-miR5021 and ath-miR5015b were predicted as the two most prominent miRNA hubs across both time points (**Figures 5a, b**), targeting several genes, including those involved in differentiation and plant reproductive processes. These predicted miRNA hubs also regulate several genes with established roles in defense and stress-associated pathways. Among the targets is *NPR3*, a salicylic acid (SA) receptor that binds SA with different affinities and functions as an adaptor for the Cullin 3 ubiquitin E3 ligase to mediate SA-regulated degradation of related receptor NPR1, thereby contributing to systemic acquired resistance in plants (Fu et al., 2012). Additional targets include the members of the Plant U-box E3 ligase gene family, such as *PUB25*, which confers freezing tolerance in plants and is highly expressed in root and internode tissues (Wang et al., 2019; Karthik et al., 2025). Other targets also included the defense and immune receptor genes, including *JAZ5* and *TIR-NBS-LRR* immune receptor genes. These hubs also target genes such as *β-1,3-glucanase (BG5)* and *GAE1*. The predicted interaction of our lncRNA with β-1,3-glucan-related genes suggests a possible role in reproductive development, as callose (β-1,3-glucan) is transiently deposited around the megaspore mother cell and developing megaspores and is removed from the functional megaspore during the transition to gametogenesis (Pinto et al., 2024). Other predicted targets, such as *Glucuronate 4-epimerases (GAEs)*, like *GAE1*, convert UDP-D-glucuronic acid to UDP-D-galacturonic acid, catalyzing a key step in pectin biosynthesis, and are essential for maintaining the structure and integrity of plant cell walls. GAE1 also contributes to pathogen resistance, as *GAE1* and *GAE6* mutants exhibit compromised disease resistance (Bethke et al., 2016).

Gene Ontology (GO) analysis revealed significant enrichment in biological processes related to adaptive and immune responses during stress and pathogen attack. Enriched GO terms included cellular response to hypoxia (GO:0071456), cellular response to decreased oxygen levels (GO:0036294), response to wounding (GO:0009611), suggesting the involvement of lncRNAs and their network in adaptive responses. Other GO terms, such as immune effector process (GO:0002252) and defense response to bacterium (GO:0042742), which are associated with immune responses, were also enriched. Overall, the predicted mRNA targets of lncRNA and miRNA hubs, together with GO term analysis, reveal a gene interaction network primarily involved in stress and defense responses, consistent with previous studies (Mondragón-Palomino et al., 2017; Kodera et al., 2021). Further GO enrichment analysis after excluding the trans-target genes of the predicted dominant hub lncRNAs (*AT3G04795* (annotated), *XLOC_00121*, *XLOC_020056*, *XLOC_017394*) also enriched similar GO terms in addition to GO terms related to the regulation of responses to biotic (GO:0002831) and external stimuli (GO:0032101), and defense responses to fungi (GO:0050832), which are also related to biotic stress response and defense pathways, like other enriched GO terms, suggesting that the observed GO enrichment was robust and was not only driven by the trans-target genes of the predicted dominant hub lncRNAs.

This study suggests that lncRNAs may contribute to the regulatory landscape underlying compatible and incompatible pollination by influencing multiple interconnected biological processes. The temporal expression patterns of lncRNAs predicted in our study indicate that lncRNA-mediated regulatory mechanisms may represent an important secondary layer of molecular control, which appears to become more pronounced as compatible pollination progresses. The predicted cis-, trans-, and lncRNA-miRNA interactions identified in this study are associated with genes implicated in jasmonate signaling (*JAZ5*), carbohydrate transport (*SWEET4*), pollen tube-associated processes, cell wall modification (*GAE1* and *β-1,3-glucanase*), and stress- and defense-related functions. The prediction of differentially expressed lncRNAs, the trans-acting interactions, lncRNA-miRNA interactions found at 60 min, suggests a potential regulatory mechanism that extends well beyond the initial pollen-stigma recognition event. The predicted lncRNA target genes have been known to be involved in pollen tube elongation, vesicular transport mechanisms, peptide signaling pathways, carbohydrate translocation, membrane-associated processes, and stress responses. In parallel, Gene Ontology enrichment analysis predicted overrepresentation of pathways related to hypoxia, wounding, immune effector processes, and defense responses. Together, these findings are consistent with the hypothesis that lncRNAs may have a regulatory influence across multiple interrelated signaling pathways, suggesting that lncRNAs may play an important role in fine-tuning the transcriptional landscape during compatible pollination through the coordinated regulation of downstream cellular processes and may modulate the balance between reproductive and defense-associated signaling during pollination. However, further experimental validation is necessary to clarify the direct mechanistic roles of specific lncRNAs and to establish their roles in compatible and incompatible pollination.

The transition of *Arabidopsis thaliana* from an ancestral self-incompatible outcrossing species to a predominantly self-compatible mating system involved multiple independent genetic events affecting the S-locus and associated regulatory pathways. Various mutations disrupting the male and female specific signaling elements have been identified in wild accessions of *A. thaliana* (Boggs et al., 2009; Kusaba et al., 2001; Nasrallah et al., 2004; Sherman-Broyles et al., 2007; Shimizu et al., 2008). Interestingly, several accessions retained full-length, expressed *SRK* genes (Shimizu et al., 2008; Tsuchimatsu et al., 2010). Interspecific crosses with *Arabidopsis halleri* showed that some European accessions of *A. thaliana*, like Wei-1 consisted of a retained female functional SI response component. This suggests that SRK and other female signaling components along with their downstream signaling pathway are still functional (Tsuchimatsu et al., 2010). Moreover, a 213-base-pair (bp) inversion in the *SCR* gene was identified in most of the European accessions. The expression of the inverted 213-bp region in Wei-1 plants restored the SI response, suggesting that disruption of *SCR* was primarily responsible for the evolutionary loss of SI response. However, in an artificial SI system of *A. thaliana* expressing *AlSCRb*, *AlSRKb*, and *AlARC1* from *A. lyrata*, overexpression of *BnGLO1* was sufficient to break self-incompatibility, demonstrating that other signaling components such as a compatibility factor *GLO1* also functions in the SI response in stigma (Kenney et al., 2020).

LncRNA transcripts generally evolve more rapidly than protein-coding genes and frequently exhibit limited sequence conservation across species. The novel lncRNAs identified here may represent lineage-specific transcripts or regulatory elements that have diverged following the transition to self-compatibility. The differential expression observed in compatible pollination, particularly at 60 min, compared with incompatible pollination in this study, suggests the possible presence of a transcriptional regulatory network rather than transcripts of non-functional remnants of an ancestral SI network. However, these hypothesis can only be confirmed using functional genomics studies.

Nevertheless, the functional significance of expressed predicted novel lncRNAs remains unclear, and their failed detection in publicly available datasets may reflect highly specific temporal, spatial, or condition-dependent expression rather than evolutionary degeneration. In this direction, future investigations should focus on integrating comparative analysis, synteny-based conservation across self-incompatible Brassicaceae species, and experimental validation to distinguish conserved functional regulators from lineage-specific or evolutionarily decaying transcripts. This will provide a clearer understanding of the extent to which ancestral SI-associated regulatory networks have been retained or lost during the evolution of self-compatibility in *A. thaliana*.

## 5 Conclusion

In this study, we predicted a high-confidence set of both novel and annotated lncRNAs expressed during compatible and incompatible pollination in *Arabidopsis thaliana* at early and late post-pollination stages. Although comparable numbers of predicted lncRNAs were detected at both time points, incompatible pollination induced a strong, time-dependent shift in lncRNA regulation, characterized by predominantly downregulated and largely stage-specific differentially expressed lncRNAs at the later stage. Predicted cis-regulatory associations of lncRNAs were relatively limited in number but exhibited strong expression concordance with neighboring protein-coding genes, suggesting tightly coordinated local regulation. In contrast, the predicted trans regulatory interactions were markedly enriched at the late stage and were driven by a small number of highly connected lncRNAs targeting genes involved in self-incompatibility, stress, and hormone-related pathways. Integration of possible miRNA-mediated regulation showed an additional regulatory layer, with predicted lncRNA-miRNA-mRNA networks at a later stage and a predominance of putative miRNA-sponge interactions. Analysis of predicted lncRNA-associated target genes showed a strong temporal specificity and coordinated transcriptional changes in global expression analysis, which shows a transition from early signaling events to broader regulatory responses during incompatible pollination.

## Data Availability

The publicly available RNA-seq dataset used in this study is available in the NCBI Sequence Read Archive (SRA) under accession number SRP154565. The scripts used for data analysis are available on GitHub under the GNU General Public License v3.0 at https://github.com/nischaypatel4/LncRNA-Analysis-Pollination-Arabidopsis. The data generated during this study have been deposited in Zenodo under the Creative Commons Attribution 4.0 International license (https://doi.org/10.5281/zenodo.21266942). Additional data supporting the findings of this study are included in the article and its **Supplementary Files**.
