# Supplementary material for: Characterization of long non-coding RNAs during compatible and incompatible pollination in Arabidopsis thaliana
Source: Front Plant Sci. 2026 Jul 29;17:1868651. doi: 10.3389/fpls.2026.1868651 (PMC13462255; doi:10.3389/fpls.2026.1868651)
Supplement: Supplementary file 1 [file SupplementaryFile1.zip › Supplementary Material/Supplementary Figure S1 and S2.docx]

**Supplementary Figure S1. Principal component analysis (PCA) of RNA-seq samples at early and late pollination time points.** (a) PCA of variance-stabilized RNA-seq expression data at t = 10 min after pollination and (b) PCA at t = 60 min after pollination. PCA was performed on variance-stabilized counts generated using the DESeq2 pipeline. Each point represents an individual biological replicate, colored by pollination conditions (Compatible and Incompatible). All biological replicates were included in the PCA visualization. Based on PCA clustering patterns and euclidean distance-based replicate consistency assessment, one diverged replicate per condition per time point was excluded from downstream differential expression analyses: 10_compatible3 and 10_incompatible4 at 10 min, and 60_compatible4 and 60_incompatible2 at 60 min. PCA was used to assess sample relatedness and consistency among biological replicates within and between pollination conditions at each time point.

**Supplementary Figure S2.** **Mean–variance relationship of transcript expression at early and late pollination time points.** Mean–variance trend analysis of RNA-seq expression data of Compatible and Incompatible pollination. Each point represents a transcript, plotted as log₁₀ (mean TPM) versus log₁₀ (variance) across biological replicates. The blue curve indicates the locally weighted regression (LOWESS) fit capturing the global mean–variance relationship. Vertical dashed lines denote commonly used TPM expression thresholds: TPM = 0.01 (black), TPM = 0.05 (orange), and TPM = 0.1 (yellow). Transcripts with very low mean expression (TPM ≤ 0.05) exhibit elevated variance relative to their mean, consistent with increased variability at low expression levels. As mean expression increases, the mean–variance relationship becomes progressively smoother, with improved coupling observed around TPM > (0.05 – 0.1). This analysis was used to inform the choice of expression thresholds for downstream analysis.
